# Paramagnetic Properties of Fullerene-Derived Nanomaterials and Their Polymer Composites: Drastic Pumping Out Effect

**DOI:** 10.1186/s11671-017-2241-3

**Published:** 2017-08-01

**Authors:** Andriy A. Konchits, Bela D. Shanina, Serhii V. Krasnovyd, Alexander I. Burya, Olga Yu Kuznetsova

**Affiliations:** 1 V.E. Lashkaryov Institute for Semiconductor Physics NAS of Ukraine, Kyiv, 03028 Ukraine; 2 Dniprodzerzhynsk State Technical University, Dniprodzerzhinsk, 51918 Ukraine; 3 Dnipropetrovsk State Agrarian and Economic University, Dnipropetrovsk, 49600 Ukraine

**Keywords:** Nanomaterials, Fullerene, Electron properties, Paramagnetic oxygen, Carbon defects, Carbon flakes

## Abstract

The evolution of paramagnetic properties of the fullerene soot (FS), fullerene black (FB), and their polymer composites Phenylon C-2/FS, FB has been studied using the electron paramagnetic resonance (EPR) method. For the first time, a drastic growth of the EPR signals in the FB, FS, and composite samples was observed under pumping out at temperatures *T* = 20 ÷ 300 °C, which is attributed to the interaction between carbon defects and adsorbed gas molecules, mainly oxygen.

It is shown that the ensemble of paramagnetic centers in the FB, FS, and the composite is heterogeneous. This ensemble consists of three spin subsystems 1, 2, and 3 related with different structural elements. The subsystems give three corresponding contributions, *L*_1_, *L*_2_ and *L*_3_, into the overall contour of the EPR signal. The most intensive and broad signal *L*_3_ is caused by 2D electrons from the surface of carbon flakes. Theoretical calculations of the *L*_3_ signal line shape were carried out, and the decay rate of the integral intensity has been obtained for each component *L*_1_, *L*_2_, and *L*_3_ after the contact of the sample with the ambient air. The signal decay process in the bulk composite samples is much slower due to their low gas permeability at room temperature (RT).

## Background

Carbon-based nanomaterials, such as graphene, nanotubes, fullerene, onion-like carbon (OLC), nano-diamond (ND), and carbon dots, attract a significant interest of the researchers over the past decade. These materials demonstrate a great variety of their sizes and structures, from small molecules to long chains, as well as variations of the sp^1^, sp^2^, and sp^3^ bond ratio [1]. The unique properties of carbon-based nanomaterials are widely used in many fields, including fundamental material science [1–3], energy [4, 5], biology and medicine [6–9], and environment [10]. Fullerenes and their derivatives occupy the important place among the nanocarbon materials, as well as nano-diamonds and carbon nano-onions (multishell fullerene-type nanostructures). A common property of the nanomaterial group is their ability of mutual transformations, for example, soot or fullerene black to OLC [11, 12], ND in OLC [13], and graphene to fullerene [14].

At present time, the practical application of fullerene materials continues to grow due to new applications in biology [6, 7], medicine [9], synthesis of nanocomposites with unique properties [15, 16], materials for electromagnetic shielding [17–20], and others. The physical-chemical properties of the fullerene-type nanomaterials depend on their electronic properties, the structural imperfection, surface area, and others. For example, the composites of onion-like carbon nanoparticles (their synthesis includes the presence of oxygen) reveal the enhanced microwave-absorption properties [18]. The existence of a huge amount of defects in the fullerene-like materials and deviation of their structure from planarity (“pyramidalization”) essentially affect their reactivity [21–24]. The EPR spectroscopy is generally used to get detailed information about the electron properties of fullerene-like materials. The structure and paramagnetic properties of the fullerene soot (FS) and fullerene black (FB) were studied in [25–28]. The EPR signal of these materials is shown to be characterized by the following parameters: *g* = 2.0022 ÷ 2.0023, Δ*H*_pp_ ≅ 2 G. The concentration of paramagnetic radicals *N*_s_~10^21^ g^−1^ and *N*_s_~3 × 10^18^ g^−1^ in the initial samples FS [25] and FB [27], has been found, respectively. These parameters are changed insignificantly at the presence of molecular oxygen, except for FB samples, of which the *N*_s_ value increases by the order of magnitude after evacuation at *T* = 150 °C [27]. The results obtained for the soot were explained using a model where the fullerene soot particles are assumed to be encapsulated in the highly defective onion-like carbon (OLC) [25].

At the same time, the effect of oxygen on the EPR intensity of radicals is very strong for FB (fullerene-free FS) [27]. The knowledge of the nature and mechanisms for the interaction of the fullerene-like materials with the molecular oxygen remains important, particularly taking into account the results obtained within the recent years [29, 30], where remarkable EPR properties of similar nanocarbon structures were shown to be related with the interaction between paramagnetic centers and gas molecules.

The main aim of our study is to clarify the nature of paramagnetic defects in the fullerene soot and fullerene black, as well as mechanisms of their interaction with molecular oxygen. Furthermore, features of these interactions and the role of polymer matrix will be studied in the composites, based on the aromatic polyamides Phenylon C-2 (PhC-2) which are characterized by strong intermolecular interactions due to hydrogen bonds. Materials like “superplastic” are promising for increase of the heat-resistance and strength in the space technologies. We have previously demonstrated that the presence of fullerene FB and FS fillers, significantly improves mechanical properties of such composites [31]. The different type of fillers is improving electronic properties of the polymer nanocomposites [32].

## Methods

Samples C60, fullerene soot, and fullerene black were obtained from NeoTechProduct (Russia, St. Petersburg) and were used as is. According to specification (http://www.neotechproduct.ru/main_page), FS was obtained by means of evaporation of graphite using the arc method. The FS samples are black powder, insoluble with the bulk density of about 0.25 g/cm^3^ and the fullerene content of about 10%. FB samples are a powder product after extraction of fullerenes from FS. Extraction is carried out with the help of nonpolar organic solvent (o-xylol) and post-treatment with steam to remove organic solvent. The fullerene C60 content in the FB samples is ≤0.3%.

The original polymer matrix PhC-2 is the linear heterocyclic copolymer containing in its macromolecule’s main chain the –HNCO– amide group linked at both sides by phenile fragments. It has been obtained by means of emulsion polycondensation of metaphenilenediamine supplemented by the mixture of dichloranhydrides of isophtale and terephtale acids which were taken in the molar ratio of 3 to 2.

The composites PhC-2/FS and FB were obtained by means of mixing the components in the rotating electromagnetic field with further treatment of the compositions by the compression molding method (*T* = 598 K, *P* = 40 МPа). The filler amount in compositions was 1.5 and 3 wt.%.

Magnetic resonance measurements were carried out at room temperature mainly using the X-band (microwave frequency *ν* ~ 9.4 GHz) EPR spectrometer Radiopan X-2244 with 100 kHz modulation of magnetic field. The estimated accuracy in the determination of the g-factor was ±2 × 10^−4^ for the observed EPR lines with linewidth Δ*H*_pp_ ≤ 10 G. The absolute accuracy of the spin density (*N*_s_) was ±50%, whereas the relative accuracy of *N*_s_ was ±20%. The paramagnetic properties of the samples were studied in the ambient air, as well as under conditions of the controlled oxygen concentration using the pumping out at *T* = 20 ÷ 170 °C. The samples were placed into a quartz tube which was evacuated at the definite temperature. Then, the samples have been put into the cavity of the spectrometer, and EPR spectrum was recorded without change of the pumping out conditions.

## Results and Discussion

**Fig. 1:**
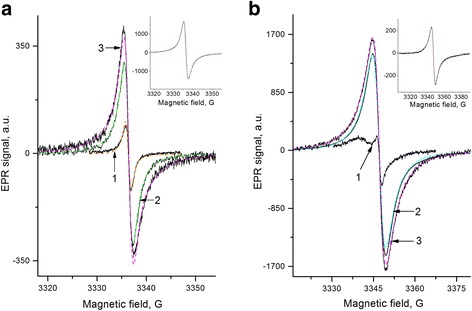
EPR spectra of fullerene, FS, FB, and composites Phenylon C-2/FS, FB. **a.** *1*—initial samples of the fullerene C60, *2*—fullerene soot (FS), *3*—fullerene black (FB) at room temperature. *Dashed lines* are the calculated signals (Table 1). *ν* = 9350 MHz. The insert: EPR spectrum of the fullerene black at *T* = 30 K. **b.** Composites Phenylon C-2 + 3% fillers, notably: *1*—fullerene C60, *2*—fullerene soot (FS), *3*—fullerene black (FB). The supplementary broad line on the spectrum (1) belongs to Phenylon C-2. *ν* = 9375 MHz, gain = ×5. The insert: The EPR spectrum of the composite PhC-2 + 3% FS at *T* = 30 K. *Dashed lines*—fitting (Table 1)

Figure 1a demonstrates the EPR spectra of fullerene C60, fullerene soot, and fullerene black at room temperature. Within the experimental error, all the spectra are characterized by g-factor *g* = 2.0024 ± 2 × 10^−4^. The line shape is the Lorentzian only for the sample C60, whereas it is described by the sum of two Lorentz lines for the FS and FB samples. The spin concentration and the contribution of individual components to the total intensity of the spectrum are shown in Table 1 for these and other samples studied. The insert in Fig. 1a shows the EPR spectrum FB sample at *T* = 30 K. Parameters of this spectrum are given also in Table 1.

**Table 1:** EPR characteristics of studied samples

Sample	*N*_s_, cm^−3a^	The contribution of the components in the total spectra intensity
Fullerene C60		
C60	~5 × 10^15^	Pure Lorentzian, Δ*H* = 0.9 G
Pump. at RT	−−//−−	No pumping out effect
Fullerene soot		
On air	~1.8 × 10^17^	0.49 [Δ*H*_1_ = 1.5 G] + 0.51 [Δ*H*_2_ = 4.2 G]
In pure O_2_	~1.5 × 10^17^	0.26 [Δ*H*_1_ = 1.3 G] + 0.74 [Δ*H*_2_ = 3 G]
RT pump.	1.7 × 10^18^	0.11 [Δ*H*_1_ = 1.2 G] + 0.89 [Δ*H*_2_ = 5.8 G]
Pump. at 150 °C	6.4 × 10^18^	0.03 [Δ*H*_1_ = 0.9 G] + 0.13 [Δ*H*_2_ = 2.4 G] + 0.84 [Δ*H*_3_ = 10 G]
Pump. at 550 °C	1.6 × 10^19^	0.59 [Δ*H*_1_ = 8.2 G] + 0.41 [Δ*H*_2_ = 22 G]
After 24 h on air	1.9 × 10^19^	0.65 [Δ*H*_1_ = 6.9 G] + 0.35 [Δ*H*_2_ = 22 G]
Fullerene black		
On air	2.4 × 10^17^	0.3 [Δ*H*_1_ = 1.6 G] + 0.7 [Δ*H*_2_ = 5.5 G]
In He gas, *T* = 30 K	~9 × 10^17^	0.32 [Δ*H*_1_ = 2.0 G] + 0.68 [Δ*H*_2_ = 6.5 G]
RT pumping	1.9 × 10^18^	0.35 [Δ*H*_1_ = 2.55 G] + 0.65 [Δ*H*_2_ = 6.0 G]
Pump. at 150 °C	8.0 × 10^18^	0.25 [Δ*H*_1_ = 2.0 G] + 0.75 [Δ*H*_2_ = 6.5 G]
Pump. at 300 °C	1.2 × 10^19^	0.006 [Δ*H*_1_ = 0.9 G] + 0.06 [Δ*H*_2_ = 3.0 G] + 0.93 [Δ*H*_3_ = 24 G]
Composite Phenylon C-2 + 3% fullerene soot		
On air	8 × 10^15^	0.39 [Δ*H*_1_ = 2.2 G] + 0.61 [Δ*H*_2_ = 6 G]
Pump. at 160 °C	3.5 × 10^16^	0.3 [Δ*H*_1_ = 2.3 G] + 0.7 [Δ*H*_2_ = 7 G]
Composite Phenylon C-2 + 3% fullerene black		
On air	1.2 × 10^16^	0.39 [Δ*H*_1_ = 3.8 G] + 0.61 [Δ*H*_2_ = 9 G]

^a^The apparent density is used for filler

Figure 1b presents the ESR spectra of composites Phenylon C-2 with 3% of C60 (1), FS (2), and FB (3) as the fillers. The insert in Fig. 1b shows the spectrum of the composite PhC-2/FB at *T* = 30 K. Parameters obtained as a result of fitting the calculated signals to the experimental one are given in Table 1. Properties of composite samples and their fillers were also studied under evacuation at different temperatures within the range of *T* = 20 ÷ 300 °C.

**Fig. 2:**
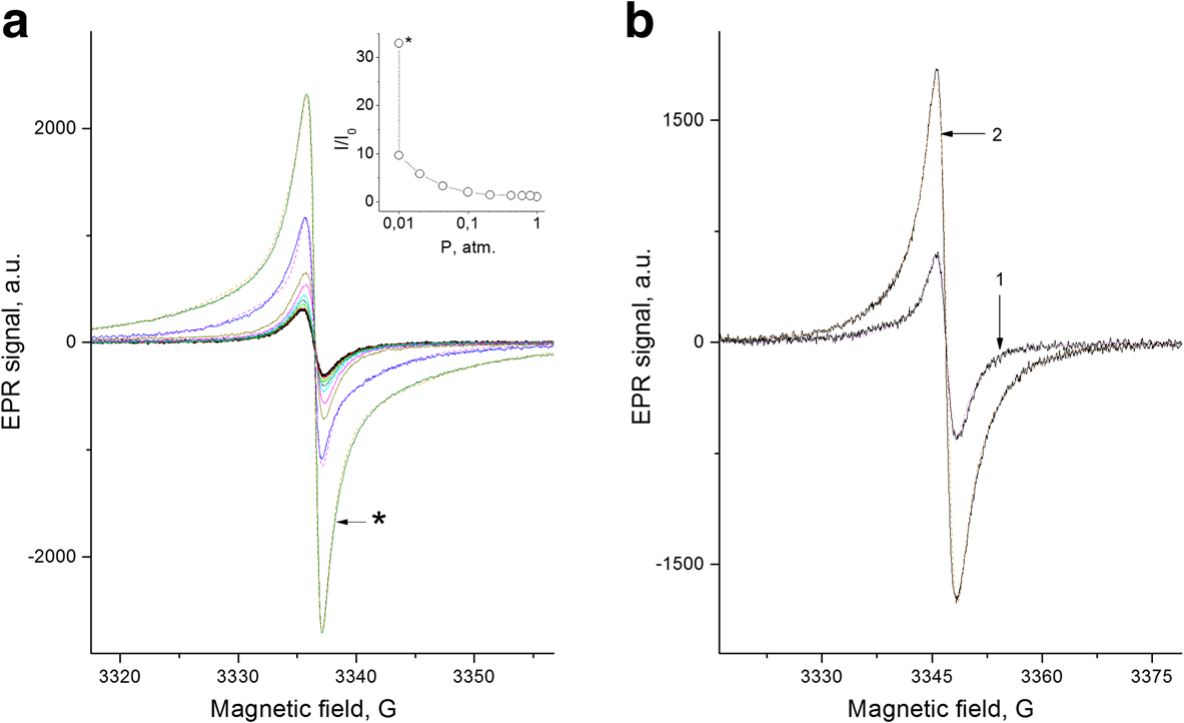
EPR spectra of fullerene soot at the various oxygen content and composite PhC-2 + 3% fullerene soot. **a.** The magnitude of the pressure of residual atmospheres (from the small to the larger amplitudes of the EPR spectrum) at room temperatures of pumping out: 1; 0.8; 0.61; 0.42; 0.21; 0.1; 0.043; 0.02; 0.001 atm., *—pump. 0.5 h at 160 *°*C, *dash line*—fitting by 3 Lorentzian. The insert: the dependence of the total intensity of the EPR spectrum on the oxygen pressure. *—pumping out 0.5 h at *T =* 160 °C. **b.** The composite PhC-2 + 3% fullerene soot (FS) before (1) and after (2) pumping out of the samples during 1 h at *T* = 160 *°*C. *Dashed lines*—fitting (Table 1)

Figure 2a shows the EPR spectra of FS in the pumping out conditions. It is seen that the intensity of the spectrum significantly increases with increasing of vacuum due to the pumping out at RT. It is obtained that the pumping out of the FS samples at higher temperatures causes a drastic increase in signal intensity by more than 30 times in comparison with the signal in the initial samples, mainly due to formation of the broad wings of the EPR spectrum (Fig. 2a, see data of fitting in Table 1). A similar effect, though not so strong, is observed in the composites after pumping out of samples at the elevated temperatures (Fig. 2b, see data of fitting in Table 1).

**Fig. 3:**
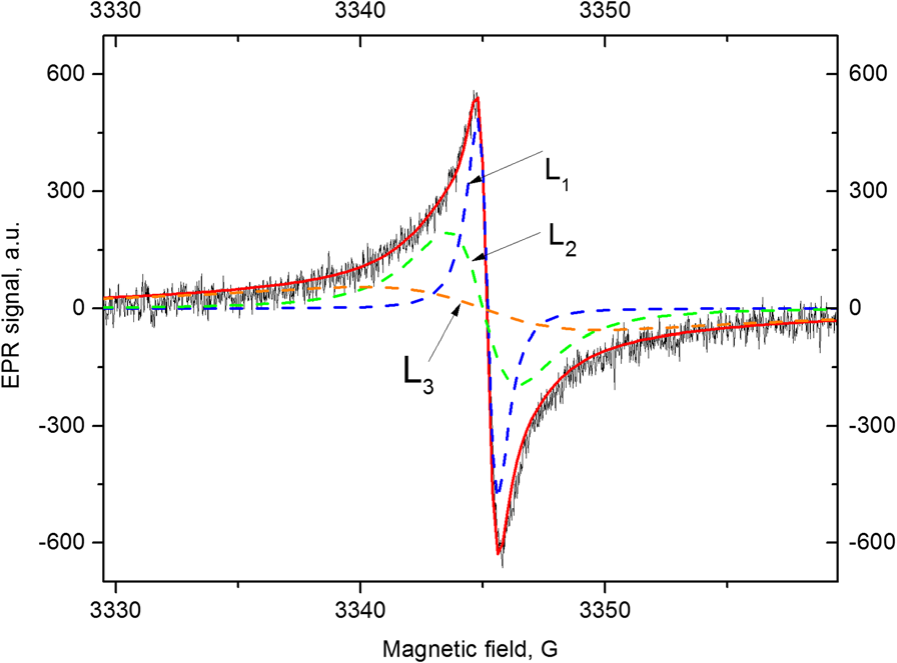
The separation of the EPR spectrum into components. The sample FB after pumping out during 0.5 h at *T* = 300 *°*C. *L*_1_, *L*_2_, and *L*_3_—components of the spectrum with *ΔH* = 0.9, 3.0, and 24 G respectively. The *solid red line* is the envelope of spectrum. *L*_3_ component was calculated taking into account subsystem of 2D electrons. *ν* = 9375 MHz

The rate of the signal recession (restoration of equilibrium) has been studied for FB sample after cessation of pumping at *T* = 300 °C and bringing the sample in contact with ambient air. This process was studied in detail for each of the three spectral components *L*_1_, *L*_2_, and *L*_3_, as shown in Fig. 3. It should be noted that the contribution of the components *L*_3_ was determined taking into account the properties of 2D electron spin subsystem (see the “Discussion” below).

**Fig. 4:**
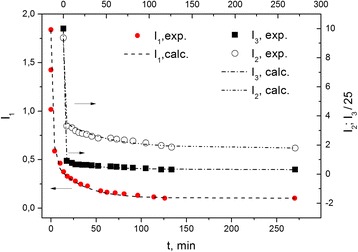
The decline of the intensity of the EPR spectrum components *L*_1_, *L*_2_, and *L*_3_ after the contact of the sample with the air. Start time *t* = 0 corresponds to the condition of the Fig. 3

The decay of these signal intensities with holding time of contacting the sample with the ambient atmosphere is shown in Fig. 4. At first, the main part of the signal decay for each component occurs for a short time (from few seconds to 1 min). Thereafter, a much slower decline (for a few hours) takes place until the recovery to the original equilibrium state of the sample.

**Fig. 5:**
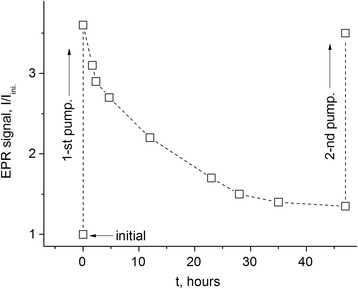
The decline of the EPR signal of the bulk composite sample PhC-2 + 3% FS (*d* ~ 1.5 × 3 × 3 mm^3^) after evacuation during 1 h at *T* = 160 *°*C. The contact of the sample was set with the environment after evacuation

A similar behavior for powder composite samples (*d* ~ 150 μ) is observed. However, it is different for the bulk samples. Figure 5 shows the time dependence of decline for the full signal intensity of the composite sample PhC-2 + 3% FS (~ 1.5 × 3 × 3 mm^3^) after pumping out at *T* = 160 *°*C and following contact with air. One can see from the comparison of Figs. 4 and 5 that the characteristic decay time of the signal intensity for the bulk composite sample is by more than one order of magnitude as large as it is the case for the powder composite and filler samples.

**Fig. 6:**
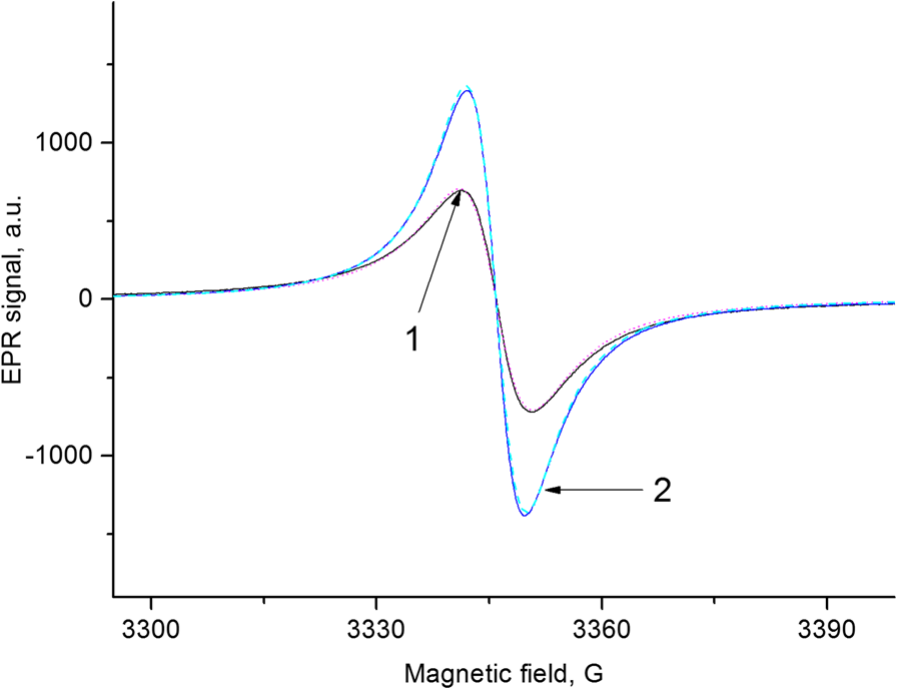
The EPR spectrum of FS sample. *1*—The sample of FS was annealed 1 h at *T* = 550 *°*C in the low vacuum, *2*—the sample of FS was stored 24 h in the ambient air after annealing

For a more detailed characterization of thermal behavior of materials under study, the annealing (in a weak vacuum) of sample FS was carried out at *T* = 550 *°*C. The recorded spectra are shown in Fig. 6. It is seen that paramagnetic properties of the annealed at *T* = 550 *°*C samples differ greatly from the properties of the unannealed samples, namely, the subsequent pumping out of the annealed sample does not lead to any drastic changes in the ESR spectrum neither in the line shape nor in the total signal intensity. Figure 6 and Table 1 show that the line shape of the spectrum is determined mainly by the Lorentzian line shape with linewidth Δ*H* = 7÷8 G in both cases, and intensity of the spectrum is almost independent of pumping. This behavior is very different from that shown in Fig. 4, as well as on the paramagnetic behavior of fullerene black samples annealed at *T* = 850 *°*C [27]. Such difference is most likely due to the fact that temperatures of 550 *°*C do not refer to the “low-temperature” but to the “mid-temperature” interval of temperature treatments of carbon materials, when their paramagnetic characteristics change significantly [33, 34]. Figure 6 illustrates this fact.

## Discussion

Paramagnetic centers in the initial FS and FB samples were observed with their concentration of 2⋅10^17^ cm^−3^ and *g*-value of 2.0024 ± 2 × 10^−4^, i.e., close to that for paramagnetic defects in many carbonaceous materials, e.g., coal [35] or graphene [36]. In case of the pumping out the FS and FB samples, particularly at the elevated temperatures, the PC concentration increases more than 30 times, up to 1.2 × 10^19^ cm^−3^, (see Fig. 2a and Table 1). Before discussing the origin of the PC and the reason for the drastic pumping out effect, let us analyze in detail the evolution of the signals and the line shape of the EPR spectrum under these conditions.

The material under study was a mixture of three-dimensional fullerene-like objects and 2D unclosed carbon flakes of various shapes. Therefore, in our analysis, we should also take into account the presence of spins localized on the open and flat objects belonging to the two-dimensional (2D) electron system.

### Contributions of 2D Spin System to the EPR Spectrum

Description of the experimental signals was carried out using the sum of two Lorentzians *L*_1_ and *L*_2_ for three-dimensional paramagnetic systems and the theoretical EPR signal *L*_3_ for the diluted 2D spin system of 2D electrons at the carbon flakes. The latter was found in [37–39] as the Fourier image of free induction decay.

1a $$ {L}_3(w)\kern0.5em =\kern0.5em {I}_0\cdot \underset{-\infty }{\overset{\infty }{\int }} \exp \Big(-{\left(t/{T}_2\right)}^{2/3} \exp \left(i\left(\omega -{\omega}_0\right)t\right)dt $$

1b $$ F\left(\omega \right)={L}_1\left(\omega \right)+{L}_2\left(\omega \right)+{L}_3\left(\omega \right), $$

The signals in (1b) are written in the order of increasing linewidth. In the experiment, the resonance signals were recorded as a derivative of the absorption signal. Therefore, the derivative of *F*(*ω*) consists of two derivatives of the Lorentz functions, which are well known, whereas the *L*_3_^’^(*ω*) is calculated as a derivative of the absorption signal (1a). The derivatives can be written as follows:

2 $$ {L}_1^{\hbox{'}}\left(\omega \right)+{L}_2^{\hbox{'}}\left(\omega \right)=-{2}^{\ast }{A_1}^{\ast }{z}_1/{\left(1+{z}_1^2\right)}^2-{2}^{\ast }{A_2}^{\ast }{z}_2/{\left(1+{z}_2^2\right)}^2,\kern0.5em \mathrm{where}\kern0.5em {z}_{1,2}=\left(\omega -{\omega}_{1,2}\right)/{\varDelta}_{1,2} $$

3 $$ {L}_3^{\hbox{'}}\left(\omega \right)=-{A_3}^{\ast }{\varDelta_3^{-1}}^{\ast}\int \exp {\left(-{x}^2\right)}^{\ast }{x}^{5\ast } \sin \left({z_3}^{\ast }{x}^3\right)dx,\kern0.5em \mathrm{where}\kern0.5em {z}_3=\left(\omega -{\omega}_3\right)/{\varDelta}_3,\kern0.5em x={t}^{1/3} $$

Integration in (3) is done numerically.

**Fig. 7:**
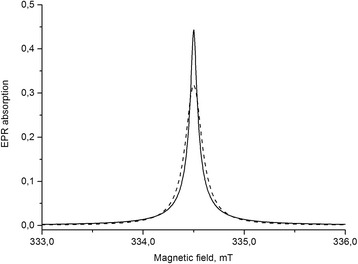
Comparison of absorption signals of the Lorentz shape (*dotted line*) with the line shape for the 2D system (*solid line*) with unit amplitudes and widths of signals

Figure 7 illustrates the comparison of the width and line shape for the Lorentzian and calculated signal from Eq. (1a). One can see that the signal for the 2D system is narrower than the Lorentzian absorption signal in the center and wider at its wings. As a result of the computation of (1a, 1b), (2), and (3) with the following fitting to the experimental spectrum, the amplitudes *A*_i_, the resonance fields *ω*_0*i*_, and the width of signals Δ_*i*_ = 1, 2, 3 have been found.

Figure 3 represents a good consistency between the experimental spectrum and the calculated spectrum including two Lorentzian derivatives plus the resonance signal of two-dimensional spin system. Therefore, after the sample pumping out, three spin subsystems contribute to the spectrum with the resonance signals *L*_1_, *L*_2_ and *L*_3_, each of which decreases with time after bringing the sample into contact with the environment (Fig. 4). The comparative contributions of three subsystems can be seen in Fig. 3 and are presented in Table 1.

Lorentzian *L*_1_ has the constant *g*-factor of 2.0024, the smallest linewidth equal to 0.9 G, and the amplitude of the signal which changes by an order of magnitude in dependence of the holding time in the air. The small width of the line *L*_1_ shows its isolation from other spin systems. A similarity of its *g*-factor and the *g*-factor of the defects in the fullerene indicates that paramagnetic centers of this subsystem belong to fullerene-like three-dimensional objects. The higher concentration of the centers responsible for the *L*_1_ signal, compared with the pure fullerene C60, should not be surprising. It is known [40] that this signal belongs to C_120_O defects, whose concentration sharply increases upon annealing the samples at temperatures of ~100 ÷ 200 °C, which is the case in our experiments. Second Lorentz signal *L*_2_ is characterized by three times larger signal width in comparison with the *L*_1_ signal and has a *g*-factor of 2.0025. The conclusion about the nature of this subsystem will be made after discussing the integral intensities and their decay with holding time in the air, *t*_air_. It is worth noting only that all the amplitudes are changed by the order of magnitude in the course of holding in the air. The *L*_3_ signal from the two-dimensional electron spin system has the greatest width and a *g*-factor of 2.0025. In contrast to other two subsystems, the *L*_3_ signal width changes from 25 to 16.5 G. The large signal width and its decrease with the decrease of spin concentration during holding time *t*_air_ suggest that the dipole-dipole interaction between the spins determines the linewidth. Its value of 20 G corresponds to the average distance between the spins of about 1 nm. Carbon flakes have a size of about 10 to 100 nm, so that each flake can contain several uncompensated spins.

The integral intensities have been obtained and their dependence versus the holding time *t*_air_ in air is shown in Fig. 4. A common feature of all the subsystems is a rapid change in the concentration of the spin on the first stage, where *t*_air_ ≤1 min and a slow concentration decline thereafter. As seen from Fig. 4, the decrease in the integral intensity of *L*_1_ signal is smoother in comparison with that for signals *L*_2_ and *L*_3_. Its main change in the air proceeds in tens of seconds, whereas further change continues for hours. The dependence of the integral intensities of the time *t*_air_ is described by two exponential functions for all signals:

$$ \begin{array}{l}{I}_1\left({t}_{\mathrm{air}}\right)=0.152\cdot \left(1+13\cdot \exp \left(-\frac{t}{2}\right)+4\cdot \exp \left(-\frac{t}{30}\right)\right);\\ {}{I}_2\left({t}_{\mathrm{air}}\right)=1.38\cdot \left(1+4\cdot \exp \left(-\frac{t}{1}\right)+1\cdot \exp \left(-\frac{t}{50}\right)\right);\\ {}{I}_3\left({t}_{\mathrm{air}}\right)=0.21\cdot \left(1+30\cdot \exp \left(-\frac{t}{0.8}\right)+1.5\cdot \exp \left(-\frac{t}{50}\right)\right).\end{array} $$

The decay time is the shortest for the two-dimensional spin system, *τ*_3_ = 0.8 min. From the analysis of the spectra, it was found that the change in the spin concentration of the two-dimensional system, as determined by the integral intensity of the *L*_3_ signal, is accompanied by the variation of signal width, whereas the width of signals *L*_1_ and *L*_2_ remains unchanged when their integral intensity, i.e., spin concentration, varies. This confirms the spin-spin mechanism for the broadening of a two-dimensional signal. The rapid initial decline of the intensities *I*_3_ and *I*_2_ is explained by the easy accessibility of these subsystems for the gas particles, e.g., oxygen, in contrast to twice a slower decline in *I*_1_ for subsystem 1, whose spins are encased in the fullerene-like three-dimensional structures and less available for diffusing particles. Spin concentration of subsystem 1 is small and corresponds to the estimated concentration of fullerene-like particles in the sample. Subsystem 2, which is open for the gas molecules and has a lower concentration compared to the 2D subsystem 3, most likely belongs to the uncompensated spins of carbon bonds of the edge atoms at carbon sheets.

### The Nature of the EPR Signals and the Role of Molecular Oxygen

The nature of paramagnetic defects of carbonaceous materials (carbon nano-onion, graphene, fullerenes and fullerene-derived materials, astra lens, and so on) has been widely discussed over the past decade [23–31, 33–36, 41]. Fullerenes can contain defects of molecules C60 [40, 42], FS, and FB—uncompleted *sp* ^2^—or *sp* ^3^ valences, e.g., the edges of carbon fragments [25–27, 36] or localized spins attributed to *sp* ^3^ dangling bonds between neighboring carbon sheets [29]. It is well known, that the molecular oxygen localized near carbon dangle bonds causes a noticeable broadening of the EPR signal. The quantitative dependence of the linewidth on the concentration O_2_ molecules was studied in detail in [35, 43]. As seen from Table 1, all fullerene-like materials do not reveal the effect of the air on the linewidth in the EPR spectra. Moreover, the widest component *L*_3_ is narrowing in the course of the long time holding in the air. Therefore, the role of the molecular oxygen in the studied here materials is quite different, if it generally exists.

Let us compare the properties of different fullerene-like samples after the pumping out as given in Table 1. The third column shows the relative contributions of three spin subsystems to the EPR spectra. It calls attention the fact that the narrowest signal *L*_1_ exists in all the samples including the pure fullerene, where *L*_1_ belongs to the only spin subsystem. This spin system is specific for the carbon dangling bonds belonging to fullerene molecule with the structural defects. The *L*_1_ contribution is not higher than 30% for all fullerene-like samples after pumping out. Signals *L*_2_ and *L*_3_ appear just after pumping out. It is worth noting that spin subsystem 2 is observed at any temperature of pumping in contrast to spin subsystem 3. It says about a difference between these systems in the binding energy between the evacuated molecules and the carbon atom. The spin subsystem 2 is characterized by the smallest binding energy, which we attribute to the edge carbon atoms at the carbon flakes. In fact, the smallest binding energy exists between carbon atoms located at the nearest flake-neighbors and it is being broken under pumping out. Spin subsystem 3 is characterized by the largest spin concentration (by 2 ÷ 3 order as large as spin concentrations for subsystems 1 and 2), which results in the largest linewidth and the line shape typical for 2D spin subsystems. This suggests that spin subsystem 3 belongs to dangling carbon bonds at the surface of carbon flakes. This subsystem is observed mainly at the pumping temperature *T* ≥ 100 °C and its contribution is larger if the pumping temperature increases up to *T* = 300 °C. It means that the binding energy for these carbon atoms with gas molecules is larger than for the edge atoms.

In our experiments, we did not observe either the broadening of *L*_1_, *L*_2_, and *L*_3_ signals caused by oxygen, or a very broad EPR line. However, recently, such EPR line with *ΔH* ≥ 200 G was observed in the oxidized graphene [44]. In [44], the narrow ESR line of carbon defects with *g* = 2.002 was gradually restored in contact with nitrogen gas, which substituted the oxygen, and this process has reached saturation after about 10 min of holding under nitrogen.

We observe also a significant change in the properties of the samples during annealing the fillers at an elevated temperature (Fig. 6 and Table 1), which indicates on the modification of the material structure. This result is consistent with the data [25, 27], where a change was also observed in the structure of the FS [25] and paramagnetic properties of FB [27] in the course of high-temperature annealing.

### EPR in the Composite Phenylon C-2/Fullerene Soot

It is seen from Fig. 1a, b, as well as in Table 1, that the width of the lines of individual components in the EPR spectrum of the composite is greater than their value for the filler. Moreover, a broader component of spectrum is dominant (0.61) in the composite, and the width value *ΔH*_2_ = 6 G is close to the value for the pumped out FS sample (Table 1). This seems to be not surprising if to take into account that the conventional cooking process of the composite takes place at *T* = 325 °C and the gas content in the melt is not controlled. The effect of pumping out the composite at *T* = 160 °C (Fig. 2b) is much weaker than it occurs separately for the filler (Fig. 2a), which is clearly associated with a much worse gas permeation in the composite in comparison with that for the filler. For the same reason, the rate of decay of the EPR signal in the composite is significantly reduced after evacuation at *T* = 160 °C, by more than an order of magnitude, if thereafter the sample is brought in contact with the air (compare Figs. 4 and 5). The fast exponent of the signal decay (~1 min) is absent (see Fig. 5), because the process is almost entirely controlled by slow rate of oxygen penetration into the sample.

## Conclusions

Fullerene soot and fullerene black actively interact with gas molecules from the environment. This leads to an almost complete (about 95%) suppression of EPR signals from carbon defects, which can be restored after pumping out the samples in the temperature range of 20 to 300 °C. Under these conditions, a complex EPR spectrum consisting of three components, each of which originated from the specific elements of the sample structure, is clearly manifested. The most powerful contribution *L*_3_ comes from the 2D electron spin subsystem at the surface of the carbon flakes. The *L*_1_ and *L*_2_ components belong to defects of fullerene (or fullerene-like) molecules and edge carbon atoms at the carbon flakes. Theoretical calculations of the *L*_3_ signal line shape were carried out and a good agreement with experiment has been obtained. The decay rate of the *L*_1_, *L*_2_, and *L*_3_ components in the total EPR signal (the restoration of equilibrium), after bringing the sample into contact with the ambient air was obtained from the comparison with the experiment.

These phenomena occur also in the bulk of composite samples Phenylon C-2/FS, FB. However, they are observed not so clear, which is possibly due to other prehistory of samples, as well as to the apparent low gas permeability of composites at RT.

It remains questionable whether the carbon dangling bonds are “killed” in contact with the adsorbed gas for the short time (*t*_air_ ~ 1 s) between the end of pumping out and the first EPR registration in the ambient air or their EPR signal becomes greatly broadened and unobservable due to the contact with paramagnetic oxygen. Finally, we believe that the highly absorbent structures, as described above, may find their use in environmental studies, as well as oxygen sensors in biomedicine.

## Abbreviations

EPR: Electron paramagnetic resonance
ESR: Electron spin resonance
FB: Fullerene black
FS: Fullerene soot
ND: Nano-diamond
*N*_s_: Spin concentration
OLC: Onion-like carbon
PC: Paramagnetic centers
PhC-2: Phenylon C-2
RT: Room temperature
